# Supplementary material for: Effect of Sn Content on the Microstructure, Mechanical Properties and Corrosion Behavior of Biodegradable Mg–x (1, 3 and 5 wt.%) Sn–1Zn–0.5Ca Alloys
Source: Materials (Basel). 2018 Nov 26;11(12):2378. doi: 10.3390/ma11122378 (PMC6316958; doi:10.3390/ma11122378)
Supplement: Supplementary file 1 [file materials-11-02378-s001.pdf]

materials

1. Supporting Figures S1-S3

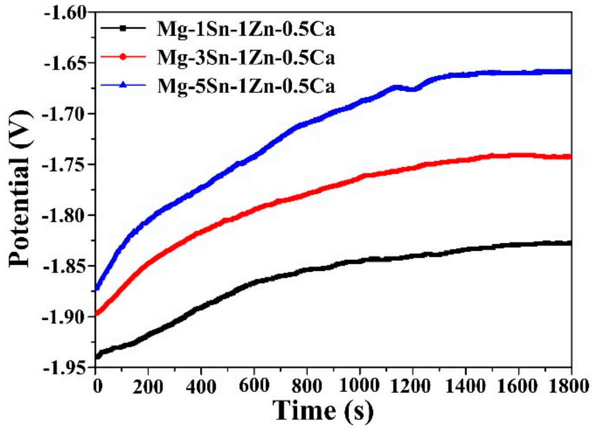

Figure S1. The open circuit potential of Mg−xSn−1Zn−0.5Ca alloys in Hank's solution.

For electrochemical tests, the samples were immersed into SBF solution at 37 °C for 1800s to obtain an approximate steady open circuit potential (OCP, V vs. SCE) before electrochemical impedance spectroscopy (EIS) test. Figure S1 shows the open circuit potential (OCP) curves of the hot-rolled Mg−xSn−1Zn−0.5Ca (x=1, 3 and 5wt.%) alloys immersed in SBF solution for 1800s. At the first 600s, the OCP gradually increased, finally, the OCP reach an approximately steady potential.

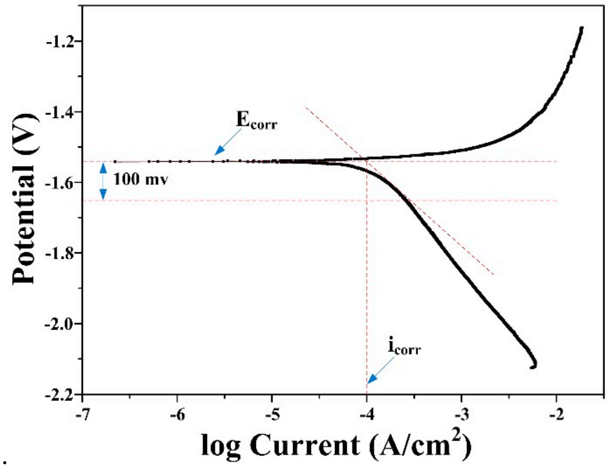

Figure S2. The schematic of method of obtaining corrosion current density.

Due to the abnormal anodic behavior that Mg and its alloys typically show negative difference effect [1,2], the corrosion current density was obtained using the cathodic branch [3]. Fig. S2 shows the schematic of method of obtaining corrosion current density. The fitted results were shown Table 4.

Table S1. Fitted EIS results of hot-rolled Mg−xSn−1Zn−0.5Ca alloys in Hank's solution.

Alloy	R_s (Ω cm²)	CPE_f ($Ω^{-1}$ cm^{-2} s^{-n})	n_f	R_f (Ω cm²)	CPE_{d1} ($Ω^{-1}$ cm^{-2} s^{-n})	n_{d1}	R_t (Ω cm²)
Mg−1Sn−1Zn−0.5Ca	36.64	$2.159×10^{-5}$	0.8671	421.7	$1.216×10^{-4}$	0.9908	1127.5
Mg−3Sn−1Zn−0.5Ca	34.95	$3.543×10^{-5}$	0.8062	270.2	$1.593×10^{-4}$	0.9305	842.1
Mg−5Sn−1Zn−0.5Ca	31.53	$3.991×10^{-5}$	0.8265	186.9	$3.67×10^{-4}$	0.8788	405.2

These experimental data can be fitted using the equivalent circuit, the equivalent circuit and the fitting data of the EIS spectra are presented in Fig. 8(d) and Table S1. The R_s, R_f and R_t represent solution resistance between the reference electrode and working electrode, charge transfer resistance and film resistance, respectively. A constant phase element, CPE_f, represents the corrosion product film capacitance, CPE_{dl} represents the capacitance of the charge transfer and double layer, n_f and n_{dl} are indices of the dispersion effects of CPE_f and CPE_{dl}, respectively. Besides, L represents the inductance and R_L represents the corresponding resistance. It is generally known that higher R_f and R_t values represent the better corrosion resistance, whereas smaller CPE_f and CPE_{dl} correspond to higher values of R_f and R_t, respectively. In the present study, R_f values of the Mg–xSn–1Zn–0.5Ca (x=1, 3 and 5wt.%) alloys were 421.7, 270.2, and 186.9 Ω cm^2 ,and the R_t values were 1127.5, 842.1, and 405.2 Ω cm^2. The R_f and R_t decreased with the addition of increasing Sn content. This means that Mg–1Sn–1Zn–0.5Ca has best corrosion resistance among Mg–xSn–1Zn–0.5Ca alloys.

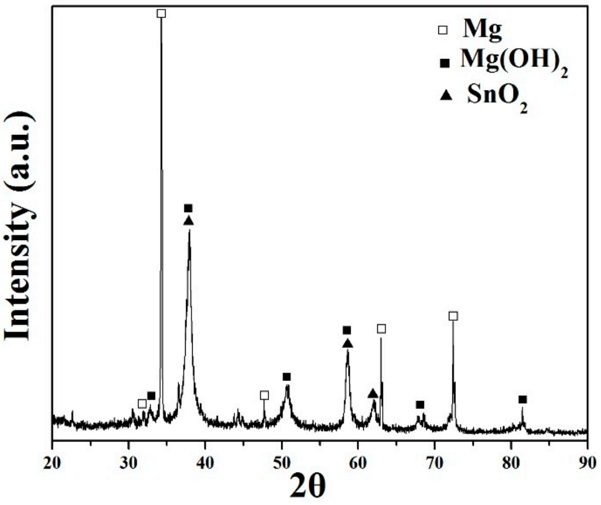

Figure S3. XRD patterns of hot-rolled Mg−5Sn−1Zn−0.5Ca alloy after being immersed in Hank's solution for 7 days.

References

1. Liao, J.; Hotta, M.; Mori, Y. Improved corrosion resistance of a high-strength Mg-Al-Mn-Ca magnesium alloy made by rapid solidification powder metallurgy. *Mater. Sci. Eng. A* **2012**, *544*, 10–20.
2. Song, G.; Atrens, A.; Dargusch, M. Influence of microstructure on the corrosion of diecast AZ91D. *Corros. Sci.* **1998**, *41*, 249–273.
3. Wu, P.; Xu, F.; Deng, K.; Han, F.; Zhang, Z.; Gao, R. Effect of extrusion on corrosion properties of Mg-2Ca-XAl (X = 0, 2, 3, 5) alloys. *Corros. Sci.* **2017**, *127*, 280–290.
